# ABO-Incompatible Adult Living Donor Liver Transplantation in the Era of Rituximab: A Systematic Review and Meta-Analysis

**DOI:** 10.1155/2019/8589402

**Published:** 2019-06-11

**Authors:** Dipesh Kumar Yadav, Yong Fei Hua, Xueli Bai, Jianying Lou, Risheng Que, Shunling Gao, Yun Zhang, Ji Wang, Qinfen Xie, Muhammad Ibrahim Alhadi Edoo, Vikram Kumar Chutturghoon, Tingbo Liang

**Affiliations:** ^1^Department of Hepatobiliary and Pancreatic Surgery, The First Affiliated Hospital, Zhejiang University School of Medicine, Hangzhou, 310003 Zhejiang, China; ^2^Department of Hepatobiliary and Pancreatic Surgery, Ningbo Medical Center Lihuili Eastern Hospital, Medical School of Ningbo University, Ningbo, 315041 Zhejiang, China; ^3^Department of Hepatobiliary and Pancreatic Surgery, The Second Affiliated Hospital, Zhejiang University School of Medicine, 88 Jiefang Road, Hangzhou, 310009 Zhejiang, China; ^4^Department of Hepatobiliary Surgery, Shulan (Hangzhou) Hospital, Hangzhou, 310000 Zhejiang, China

## Abstract

**Aim:**

The primary aim of this study is to compare the short- and long-term outcomes between ABO-incompatible (ABOi) adult living donor liver transplantation (ALDLT) with rituximab prophylaxis and ABO-compatible (ABOc) ALDLT.

**Background:**

The strategy of ABOi liver transplantation (LT) was originated initially to increase the donor pool and to enable liver transplantation in emergency conditions. However, ABOi ALDLT remains a controversial approach in comparison to ABOc ALDLT.

**Methods:**

PubMed, Embase, and the Cochrane Library study search were accomplished to recognize studies comparing ABOi and ABOc ALDLT. Meta-analyses were conducted based on the evaluation of heterogeneity using a fixed-effect model and a random-effect model to assess the short- and long-term outcomes following ABOi ALDLT with rituximab prophylaxis.

**Results:**

Nine studies comprising a total of 3,922 patients (ABOi = 671 and ABOc = 3,251) were identified. There was no significant difference between ABOi and ABOc groups for 1-year, 3-year, and 5-year OS and graft survival, respectively. Moreover, 1-year and 3-year OS and DFS were similar between both groups for HCC patients. However, ABOi ALDLT had higher incidences of CMV infection, AMR, overall biliary complications, and biliary stricture than ABOc ALDLT and had other comparable postoperative complications.

**Conclusion:**

Our meta-analysis included studies comparing ABOi and ABOc ALDLT after the introduction of rituximab in a desensitization protocol for ABOi ALDLT. The results of ABOi ALDLT were comparable with those of ABOc ALDLT. However, biliary complications, CMV infection, and AMR remain a concern in the era of rituximab.

## 1. Introduction

Liver transplantation (LT) has now become an ideal treatment option for patients with liver cancer and end-stage liver diseases [1, 2]; however, its use is restricted due to a limited donor pool [3, 4]. In past decades, different attempts and breakthroughs have been made to increase the donor pool [5]. One of them is living donor liver transplantation (LDLT); this applies both for urgent and elective LT [5, 6]. Moreover, in the shortage of ABO-compatible (ABOc) donors and to increase the donor pool, ABO-incompatible (ABOi) LT remains the only option for many with a rapidly worsening liver function or for one who remains on a long waiting list [7, 8]. The liver is considered as an immune-privileged organ since it has a low incidence of humoral rejection unlike the kidney and the heart [9, 10]. Taking this into consideration, different innovative B cell desensitization protocols, such as the use of total plasma exchange (TPE), double-filtration plasmapheresis, local graft infusion therapy (LGIT), splenectomy, rituximab, mycophenolate mofetil (MMF), and intravenous immunoglobulin G (IVIG), have been used to breach the blood group barrier leading to significant advancements in the outcome of ABOi [11, 12]. Thus, ABOi is no longer contemplated as a contraindication for LT.

ABOi LDLT pediatric patients are considered safe and with acceptable results probably because of their immature immune system [11, 13, 14]. However, the safety of ABOi adult LDLT (ALDLT) is debatable among the transplant community due to different risks associated to it, especially earlier graft loss, acute cellular rejection (ACR), antibody-mediated rejection (AMR), and vascular and biliary complications, compared to those associated to ABOc ALDLT [15–17]. Likewise, hepatocellular carcinoma (HCC) recurrence after ABOc ALDLT remains another major concern due to the patient's immunosuppressed state [18]. An effective desensitization protocol for ABOi ALDLT is very demanding. The introduction of rituximab, an anti-CD20 monoclonal antibody, to the desensitization protocol has brought about a significant reduction in the incidence of AMR and has improved the outcome of ABOi ALDLT [12, 19, 20]. Rituximab acts on the CD20 antigen present on B cells, thus reducing the production of B cells which are mainly responsible for acute rejection and AMR [21, 22]. Monteiro et al. [23] were the first to report the case of rituximab use in ABOi LT in 2003. Since then, there have been several studies that have reported on rituximab prophylaxis in ABOi ALDLT [22, 24–31].

To our knowledge, no systematic evaluations have been performed to determine the effectiveness and safety of the rituximab regimen in ABOi ALDLT. This study is aimed at comparing the short- and long-term outcomes between ABOi ALDLT with rituximab prophylaxis and ABOc ALDLT. Additionally, this meta-analysis also intended to assess the long-term outcomes of HCC patients following ABOi ALDLT with rituximab prophylaxis compared to those of HCC patients following ABOc ALDLT.

## 2. Methods

### 2.1. Search Strategy

Qualified studies for this systematic review and meta-analyses were selected following the earlier settled convention with the PubMed/MEDLINE, Embase, and Cochrane Library databases by two authors (DY and YFH), using a combination of the following Medical Subject Headings (MeSH) and non-MeSH terms: liver transplantation, ABO-incompatible liver transplantation, ABO-compatible liver transplantation, hepatocellular carcinoma, tumor recurrence, primary liver carcinoma, and HCC. Additionally, the pertinent bibliography lists of articles were taken into consideration to distinguish other important studies. After preliminary screening, duplicate articles, abstracts, or unpublished studies were ruled out. The Preferred Reporting Items for Systematic Reviews and Meta-Analysis (PRISMA) guidelines were used to perform this meta-analysis [32].

### 2.2. Study Selection

We considered both retrospective and prospective studies eligible for this meta-analysis with respect to the outcomes. Additionally, considering the outcome goals and ensuring the quality of this meta-analysis, we only considered fully published studies and excluded studies with only abstracts. Additionally, we designed the following predefined eligibility criteria for the selection of studies with at least one outcome of interest.

#### 2.2.1. Inclusion Criteria

1. The study should have a definition of ABOi ALDLT and ABOc ALDLT. ABOi ALDLT includes the following donor-to-recipient combinations: A to B and O; B to A and O; and AB to A, B, and O. Other combinations are regarded as ABOc, including the ABO-identical blood group
2. The study should contain ALDLT and should compare short- and long-term results between ABOi ALDLT and ABOc ALDLT
3. The study should have sufficient data to conduct a meta-analysis
4. Adult participants (>16 years of age).

#### 2.2.2. Exclusion Criteria

1. A study without human subjects
2. A study with pediatric patients and deceased donor liver transplant
3. A study containing advanced disease stage or extrahepatic metastases
4. A study with no comparison between ABOi ALDLT and ABOc ALDLT
5. A study with a multiorgan transplant
6. A study with older patients above 70 years
7. A study with duplicate data from the same institution
8. Publications such as review articles, editorials, case reports, conferences, and letters

### 2.3. Data Extraction

All data were extracted according to the study selection criteria and were abstracted in a systematized data abstraction form using Microsoft Excel 2007 (Microsoft Corp.). The extracted data included the first author, study characteristics (publication year, country, and study design), participant characteristics (average age of the recipients, sample size of ABOi and ABOc ALDLT, pretransplant MELD score, disease characteristics, pretransplant AFP level for HCC patients, number and size of tumors for HCC patients, pretransplant therapies, hospital stay, and the duration of follow-up), and outcomes (biliary complications, infectious complications, vascular complications, acute cellular rejection, antibody-mediated rejection (AMR), graft survival, overall survival (OS), and disease-free survival (DFS) for HCC patients). Moreover, in case of insufficient data, investigators were approached to collect more relevant results. Conflicts in data extraction were resolved by discussion or consensus with a 3rd reviewer.

### 2.4. Quality Assessment

The quality of included studies was evaluated with the Newcastle-Ottawa scale (NOS) [33]. The scale comprises 3 assessment factors: (1) assessment of a selection of the study groups; (2) comparability of the 2 groups; and (3) outcome assessment. The NOS ranges from 0 to 9. Studies with scores of 7 points and above were considered to be of high quality, those with 4-6 points were considered to be of moderate quality, and those with less than 4 points were considered to be of lower quality (Supplementary Table 1).

### 2.5. Statistical Analysis

All results are accounted for as in the original articles and were double-checked. A meta-analysis was carried out with RevMan Version 5.3 (Review Manager, Copenhagen: The Nordic Cochrane Center, The Cochrane Collaboration, 2014). Outcomes are calculated as pooled odds ratios (ORs) and standard mean difference (SMD) with corresponding 95% confidence intervals (CIs). Fixed-effect or random-effect models were utilized to compute summary estimates based on the evaluation of heterogeneity. Overall effects were evaluated by utilizing the *Z*-test, and heterogeneity was tested by using Cochran's *χ* ^2^ test. The *I* ^2^ statistic was utilized to evaluate heterogeneity, which was characterized as low, moderate, or high with*I* ^2^esteemed at >25%, >50%, and >75%, respectively [34]. Two-sided *P* values less than 0.05 were considered significant.

## 3. Results

### 3.1. Study Search and Included Studies

**Figure 1:**
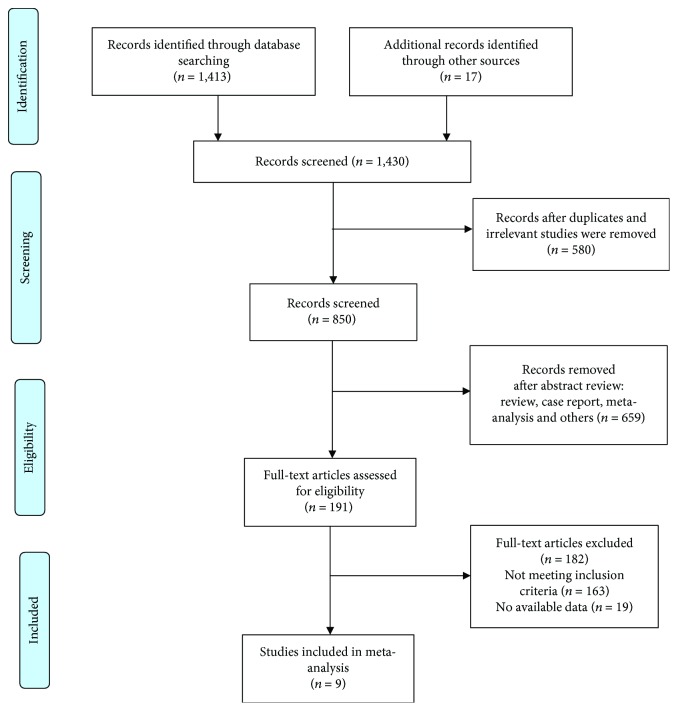
Preferred reporting items for systematic review and meta-analysis study flow diagram for literature search.

The database scans recognized 1,430 references for assessment (Figure 1), and 191 full-text articles were assessed for eligibility. Furthermore, 182 articles were excluded (articles that did not meet the inclusion criteria (*n* = 163) and those with insufficient data (*n* = 19)). The remaining 9 retrospective studies between 2015 and 2018 were eligible according to the inclusion criteria and were included in this meta-analysis, with a total of 3,922 patients (*ABOi* = 671 and *ABOc* = 3,251) (Table 1) [22, 24–31]. Although we identified 9 studies for inclusion in the analysis, two of the studies (study nos. 1 and 2) [25, 26] identified were from the same institutions in Korea as those of study nos. 3 and 4 [27, 28]. These two studies were only identified to calculate the outcome of interest for ABOi ALDLT in HCC patients and were not used for other calculations in this meta-analysis.

## 4. Meta-Analysis

### 4.1. Primary Outcome

#### 4.1.1. Patients' Preoperative and Perioperative Outcomes

**Figure 2:**
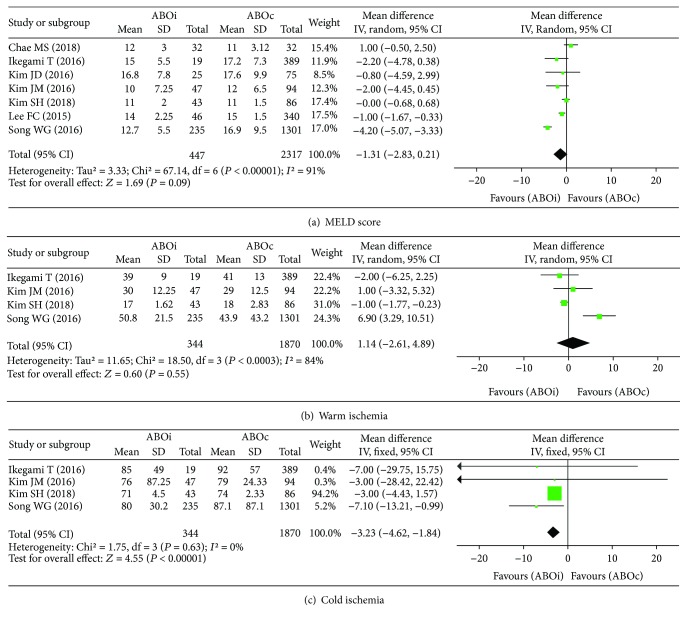
Forest plot of patients' preoperative and perioperative outcomes: (a) MELD score, (b) warm ischemia, and (c) cold ischemia.

Meta-analyses of preoperative and perioperative outcomes are shown in Figure 2. To assess the outcome measurement of the MELD score, a total of 2,764 patients were incorporated in 7 studies [22, 24, 27–31]. The*χ* ^2^test (*P* < 0.00001and*I* ^2^ = 91%) and meta-analysis using a random-effect model revealed that there was no significant difference in the MELD score between the ABOi and ABOc groups (SMD: -1.31, 95% CI: -2.83 to 0.21, *P* = 0.09, Figure 2(a)).

After classifying the data according to ischemia type, i.e., warm ischemia and cold ischemia, a meta-analysis using a random-effect model revealed that there was no significant difference in warm ischemia time (SMD: 1.14, 95% CI: -2.61 to 4.89, *P* = 0.55, Figure 2(b)) [22, 27, 28, 31] between the ABOi and ABOc groups. However, a meta-analysis using a fixed-effect model revealed that cold ischemia time was significantly shorter in the ABOi group than in the ABOc group (SMD: -3.23, 95% CI: -4.62 to -1.84, *P* < 0.00001, Figure 2(c)) [22, 27, 28, 31].

#### 4.1.2. Postoperative Short-Term Outcomes

**Figure 3:**
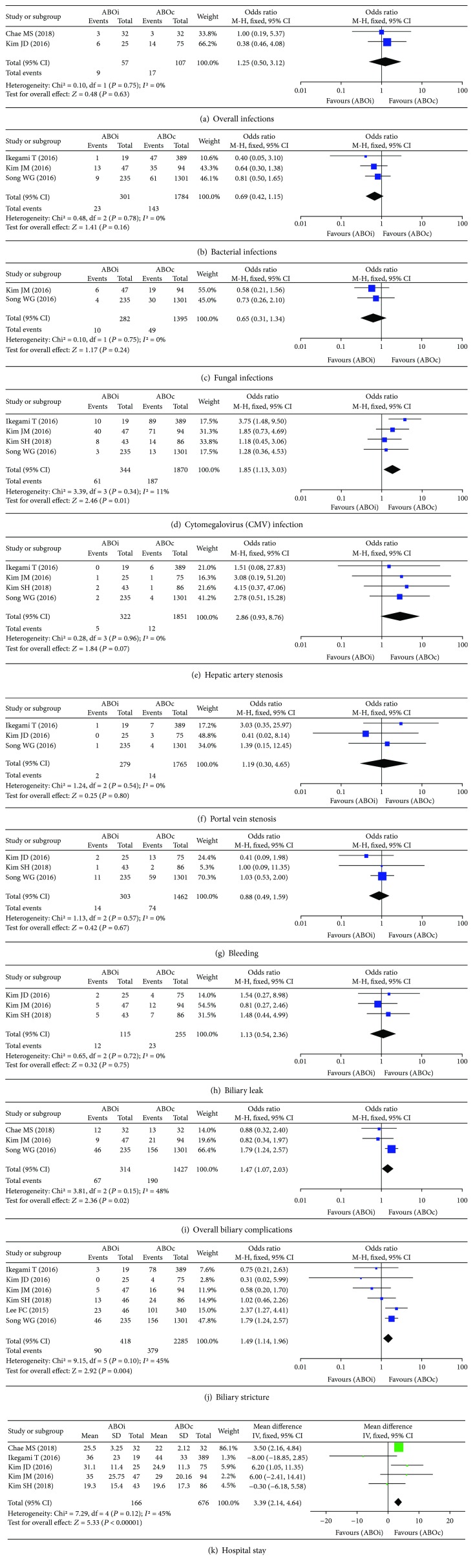
Forest plot of postoperative short-term outcomes: (a) overall infections, (b) bacterial infections, (c) fungal infections, (d) *Cytomegalovirus* (CMV) infection, (e) hepatic artery stenosis, (f) portal vein stenosis, (g) bleeding, (h) biliary leak, (i) overall biliary complications, (j) biliary stricture, and (k) hospital stay.

Meta-analyses of postoperative short-term outcomes, i.e., infectious complications, vascular complications, hospital stay, and biliary complications, are shown in Figure 3.

*(1) Infectious Complications*. Under subgroup analysis, overall infections, bacterial infections, fungal infections, and cytomegalovirus (CMV) infections were taken under consideration for meta-analysis.

A meta-analysis using a fixed-effect model revealed that there was no significant difference between the ABOi and ABOc groups for overall infections (OR: 1.25, 95% CI: 0.50 to 3.12, *P* = 0.63, Figure 3(a)) [24, 29], bacterial infections (OR: 0.69, 95% CI: 0.42 to 1.15, *P* = 0.16, Figure 3(b)) [27, 28, 31], and fungal infections (OR: 0.65, 95% CI: 0.31 to 1.34, *P* = 0.24, Figure 3(c)) [27, 28], respectively. However, a meta-analysis using a fixed-effect model revealed that CMV infection was significantly higher in the ABOi group than in the ABOc group (OR: 1.85, 95% CI: 1.13 to 3.03, *P* = 0.01, Figure 3(d)) [22, 27, 28, 31].

*(2) Vascular Complications*. Under subgroup analysis, hepatic artery stenosis, portal vein stenosis, and bleeding were taken under consideration for meta-analysis. A meta-analysis using a fixed-effect model revealed that there was no significant difference between the ABOi and ABOc groups for hepatic artery stenosis (OR: 2.86, 95% CI: 0.93 to 8.76, *P* = 0.07, Figure 3(e)) [22, 27, 29, 31], portal vein stenosis (OR: 1.19, 95% CI: 0.30 to 4.65, *P* = 0.80, Figure 3(f)) [27, 29, 31], and bleeding (OR: 0.88, 95% CI: 0.49 to 1.59, *P* = 0.67, Figure 3(g)) [22, 27, 29], respectively.

*(3) Biliary Complications*. After classifying data according to biliary complication types, i.e., overall biliary complications, biliary leakage, and biliary stricture, a meta-analysis revealed that there was no significant difference between the ABOi and ABOc groups for biliary leakage (OR: 1.13, 95% CI: 0.54 to 2.36, *P* = 0.75, Figure 3(h)) [22, 28, 29]. However, overall biliary complications (OR: 1.47, 95% CI: 1.07 to 2.03, *P* = 0.02, Figure 3(i)) [24, 27, 28] and biliary stricture (OR: 1.49, 95% CI: 1.14 to 1.96, *P* = 0.004, Figure 3(j)) [22, 27–31] were significantly higher in the ABOi group than in the ABOc group.

*(4) Hospital Stay*. To assess the outcome measurement of hospital stay, a total of 842 patients were incorporated in 5 studies [22, 24, 28, 29, 31]. The*χ* ^2^test (*P* = 0.12and*I* ^2^ = 45%) and meta-analysis using a fixed-effect model revealed that hospital stay was significantly longer in the ABOi group than in the ABOc group (SMD: 3.39, 95% CI: 2.14 to 4.64, *P* < 0.00001, Figure 3(k)).

#### 4.1.3. Postoperative Long-Term Outcomes

**Figure 4:**
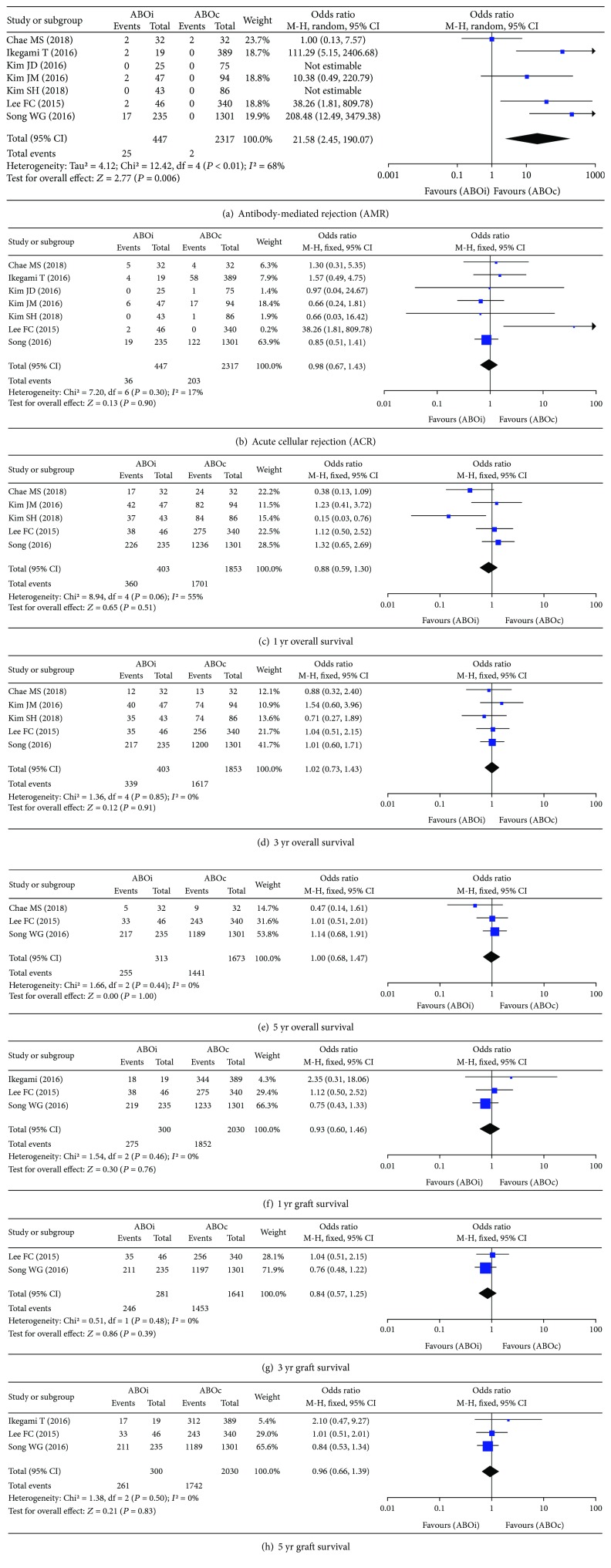
Forest plot of postoperative long-term outcomes: (a) antibody-mediated rejection (AMR), (b) acute cellular rejection (ACR), (c) 1 yr overall survival, (d) 3 yr overall survival, (e) 5 yr overall survival, (f) 1 yr graft survival, (g) 3 yr graft survival, and (h) 5 yr graft survival.

Meta-analyses of postoperative long-term outcomes, i.e., graft rejection, overall survival (OS), and graft survival, are shown in Figure 4.

*(1) Graft Rejection*. After classifying data according to graft rejection types, i.e., antibody-mediated rejection (AMR) and acute cellular rejection (ACR), a meta-analysis using a random-effect model revealed that AMR was significantly higher in the ABOi group than in the ABOc group (OR: 21.58, 95% CI: 2.45 to 190.07.13, *P* = 0.006, Figure 4(a)) [22, 24, 27–31]. However, a meta-analysis using a fixed-effect model revealed that there was no significant difference in ACR between the ABOi and ABOc groups (OR: 0.98, 95% CI: 0.67 to 1.43, *P* = 0.90, Figure 4(b)) [22, 24, 27–31].

*(2) Overall Survival (OS)*. To assess the outcome measurement of overall survival, data were classified according to 1-year, 3-year, and 5-year OS, respectively. A meta-analysis revealed that there was no significant difference between the ABOi and ABOc groups for 1-year (OR: 0.88, 95% CI: 0.59 to 1.30, *P* = 0.51, Figure 4(c)) [22, 24, 27, 28, 30], 3-year (OR: 1.02, 95% CI: 0.73 to 1.43, *P* = 0.91, Figure 4(d)) [22, 24, 27, 28, 30], and 5-year ( OR: 1.00, 95% CI: 0.68 to 1.47, *P* = 0.13, Figure 4(e)) [24, 27, 30] OS, respectively.

*(3) Graft Survival*. To assess the outcome measurement of graft survival, data were classified according to 1-year, 3-year, and 5-year graft survival, respectively. A meta-analysis revealed that there was no significant difference between the ABOi and ABOc groups for 1-year (OR: 0.93, 95% CI: 0.60 to 1.46, *P* = 0.76, Figure 4(f)) [27, 30, 31], 3-year (OR: 0.84, 95% CI: 0.57 to 1.25, *P* = 0.39, Figure 4(g)) [27, 30], and 5-year (OR: 0.96, 95% CI: 0.66 to 1.39, *P* = 0.83, Figure 4(h)) [27, 30, 31] graft survival, respectively.

#### 4.1.4. Outcome for ABOi ALDLT for Patients with HCC

Meta-analyses of the outcome for ABOi ALDLT for patients with HCC are shown in Supplementary Figure 1. To assess the outcome measurement of ABOi ALDLT for patients with HCC, a total of 1,158 patients were incorporated in 2 studies [25, 26]. A meta-analysis using a fixed-effect model revealed that there was no significant difference in preoperative AFP level (SMD: -5.96, 95% CI: -238.26 to 226.34, *P* = 0.96, Supplementary Figure 1(a)) between the ABOi and ABOc groups for patients with HCC. However, the preoperative MELD score was significantly lower in the ABOi group than in the ABOc group for patients with HCC (SMD: -1.13, 95% CI: -1.88 to -0.38, *P* = 0.003, Supplementary Figure 1(b)).

A meta-analysis of pretransplant tumor characteristics found that the maximum tumor diameter was significantly smaller in ABOi LDLT than in ABOc ALDLT (SMD: -0.30, 95% CI: -0.56 to -0.03, *P* = 0.03, Supplementary Figure 1(c)). However, the number of tumors was not significantly different among both groups (SMD: -0.22, 95% CI: -1.15 to 1.58, *P* = 0.76, Supplementary Figure 1(d)). There were no useful data for the meta-analysis of tumor size > 3 cm or tumor nodules more than 3.

To assess the outcome measurement of overall survival (OS) for HCC patients, data was classified according to 1-year and 3-year OS, respectively. There were no useful data to calculate 5-year OS. A meta-analysis revealed that there was no significant difference between the ABOi and ABOc groups for 1-year (OR: 1.31, 95% CI: 0.67 to 2.56, *P* = 0.43, Supplementary Figure 1(e)) and 3-year (OR: 1.17, 95% CI: 0.76 to 1.80, *P* = 0.48, Supplementary Figure 1(f)) OS, respectively. Furthermore, there were no data available to calculate OS stratified according to the Milan criteria.

To assess the outcome measurement of disease-free survival (DFS), data were classified according to 1-year and 3-year DFS, respectively. There were no useful data to calculate 5-year DFS. A meta-analysis revealed that there was no significant difference between the ABOi and ABOc groups for 1-year (OR: 1.26, 95% CI: 0.76 to 2.09, *P* = 0.37, Supplementary Figure 1(g)) and 3-year (OR: 1.08, 95% CI: 0.74 to 1.59, *P* = 0.68, Supplementary Figure 1(h)) DFS, respectively. Furthermore, classifying data according to the Milan criteria, a meta-analysis revealed that there was no significant difference between ABOi and ABOc groups for 1-year (OR: 0.55, 95% CI: 0.27 to 1.10, *P* = 0.09, Supplementary Figure 1(i)) and 3-year (OR: 0.22, 95% CI: 0.01 to 3.50, *P* = 0.28, Supplementary Figure 1(j)) DFS beyond the Milan criteria. There were no useful data to calculate 5-year DFS beyond the Milan criteria. Moreover, there were also no useful data to calculate DFS within the Milan criteria.

## 5. Discussion

In spite of the colossal prospect of growing the donor pool through ABOi LDLT, the safety of ABOi ALDLT is debatable among the transplant community due to poor results in the recipients such as earlier graft loss, acute cellular rejection (ACR), antibody-mediated rejection (AMR), vascular complications, and biliary complications when compared to those of ABOc ALDLT [15–17]. The utilization of ABOi living donor is an alluring answer for growing the liver donor pool, and different novel procedures for the desensitization of ABO incompatibility have yielded promising outcomes [11, 12]. However, earlier studies such as those not using rituximab in the desensitization protocol followed by ABOi LDLT showed inferior graft survival and patient survival compared to those of ABOc LDLT [15]. Nonetheless, the introduction of rituximab to the desensitization protocol has brought about significant improvements in the outcomes of ABOi LDLT [12, 19, 20].

ABOi LDLT in pediatric patients is considered safe and with acceptable results, probably because of their immature immune system [11, 13, 14]. Egawa et al. found that the 5-year patient survival rate was significantly higher in infants than in adults (85% vs. 52%) [17]. Similarly, several other studies found poor outcomes of ABOi LDLT in adults [15, 16]. Thus, ABOi LDLT in adults seems to be controversial to many.

An effective desensitization protocol is the Achilles' heel of ABOi ALDLT. However, the standard desensitization protocol for ABOi ALDLT is yet to be implemented. Most of the centers use their own desensitization protocol with or without rituximab [15]. Thus, in the scenario of conflicting results from different studies, the most important attention regarding ABOi ALDLT is graft survival, posttransplant complications, and patient survival rate following ABOi ALDLT. A standard desensitization protocol should be designed by taking both the benefits and risks into consideration. Before the era of rituximab, the high incidence of early graft loss due to AMR was the major concern of ABOi LT [12, 35, 36]. However, the incidence of AMR decreased from 23.5% to 6.2% after the introduction of rituximab, as reported by a multicenter study from Japan [12].

To date, few systematic reviews and meta-analyses have been conducted comprehensively to analyze the short-term and long-term outcomes of ABOi and ABOc LT. However, an earlier meta-analysis was reported that was not specific to ABOi ALDLT after the introduction of rituximab in the desensitization protocol. Our meta-analysis includes nine relatively high-quality studies conducted between 2015 and 2018, all containing ALDLT using rituximab in the desensitization process for ABOi ALDLT, with a total of 3,858 patients (ABOi = 639 and ABOc = 3,219); thus, we believe it is the first study of its type.

In our meta-analysis, there was no significant difference between the ABOi and ABOc ALDLT groups in terms of 1-, 3-, and 5-year graft survival and overall survival. As reported by Egawa et al. [12], the significant reduction in the incidence of AMR after the introduction of rituximab might be the cause of the improvement in graft survival of ABOi ALDLT. Moreover, the largest single-center study by Song et al. [27] also reported similar outcomes in their study. Currently, there are no definitive answers as to why the overall survival of ABOi group did not differ from the ABOc group. Previously, some studies stated that the higher MELD score was the risk factor for patient survival after LT [17, 20, 27]. However, when we looked for a MELD score between the ABOi and ABOc groups, our meta-analysis did not find any significant difference between both groups.

The incidences of postoperative complications were comparable between both groups. However, ABOi ALDLT had higher incidences of CMV infection, AMR, overall biliary complications, and biliary stricture than adult ABOc ALDLT. The possible cause of the higher incidence of CMV might be because of the immunocompromised state due to rituximab. Rituximab suppresses different stages of B cell differentiation leading to a rapid decrease in the peripheral B cell population within 48-72 hours, but which can last for several months[27, 37, 38]. Furthermore, repeated dosing of rituximab induces prolonged hypogammaglobulinemia which has a high risk for serious infectious complications [27, 38]. Likewise, in our meta-analysis two [27, 31] out of four studies reporting on CMV infection have used splenectomy in their desensitization protocol. Studies have shown that splenectomy is associated with a higher rate of serious infectious complications including CMV infection in LDLT [39]. Thus, a repeated dose of rituximab and inclusion of splenectomy in the desensitization protocol should be considered carefully. However, rituximab has additionally supplanted the need of splenectomy to prevent a posttransplant rebound increase of isohemagglutinins (IHs) [27, 40].

Despite the fact that after the introduction of rituximab to the desensitization protocol, the incidence of hepatic necrosis caused by AMR has disappeared, diffuse intrahepatic biliary stricture (DIHBS), which is a modest type of AMR, still remains to be the concern in ABOi ALDLT[19, 27]. Moreover, in the study by Song et al., DIHBS was reported to be solely in patients undergoing ABOi ALDLT [27]. The adequate reduction of B cells and the elimination of serum IH titers are important steps concerning the prevention of AMR[30]. In instances of AMR, IHs initiate the immune response by binding to the graft vessels leading to the activation of the complement system and inflammation, which may further lead to hepatic artery thrombosis and necrosis of the liver [36, 41]. Since ABO antigens are present on the bile duct epithelium, the activation of the immune response contributes to the increased incidence of uncompromising and continuous intrahepatic bile duct injury with ABOi LT [41]. As discussed earlier, rituximab, being an anti-CD20 monoclonal antibody, can suppress the activated B cell population in circulation through antibody-dependent cell-mediated cytotoxicity, direct antigen antibody reaction, and complement-dependent cytotoxicity; however, it is unable to suppress stem cells and plasma cells [42, 43]. Interestingly, plasma B cells only get triggered after they encounter allografts after LT [30]. Moreover, it has also been reported that some B cells may rescue themselves preoperatively at the time of rituximab treatment and later can get activated after LT that produces antibodies [30]. Although rituximab may thoroughly control AMR over ABO barriers, it does not perform as such on the ground, so that it cannot annihilate plasma cells that are present on the epithelium of the bile ducts, thus leading to DIHBS and biliary stricture [31, 43]. This explains why ABOi ALDLT has a higher incidence of AMR, overall biliary complications, and biliary stricture. However, our meta-analysis showed that the ABOi group had a significantly shorter cold ischemia time than the ABOc group; the reason might be due to the concern of transplant surgeons to reduce the incidence of the ischemic type of biliary stricture. Nevertheless, this has not shown to improve the incidence of biliary stricture or overall biliary complications in the ABOi ALDLT group. Previously, some of the studies have outlined that the rise in posttransplant donor-specific antibody (DSA) titers is significantly associated with the incidence of AMR; therefore, an association of DSA should also be taken into consideration as the cause of AMR [44–46]. In our understanding, the most important key to avoid AMR in ABOi ALDLT is the inhibition of newly produced antibodies. TPE is a standard procedure to decrease DSA titers, yet the titer required to avoid AMR is not well characterized [47]. Furthermore, the dosing and timing of rituximab is also a concern regarding AMR [12, 27]. Egawa et al. [12] reported that consistent single doses of rituximab (500 mg/m^2^ or 375 mg/m^2^) had a lower frequency of AMR than a single low dose (300 mg/m^2^). In the context of comparison between TPE and rituximab, Kozaki et al. [48] found that rituximab was not sufficient for decreasing antibody titers after ABOi LDLT, and TPE remains to be a mainstay of treatment for such patients. On the other hand, Kim et al. [22] concluded that desensitization using rituximab and IVIG without TEP for ABOi LDLT was safe and effective in achieving sufficient desensitization with comparable outcomes.

Furthermore, a few case reports and series have been divulged in regard to the utilization of plasma cell depleting agents, such as bortezomib, in the treatment and prevention of AMR related with the anti-HLA antibody [49, 50]. Bortezomib specifically prompts apoptosis among plasma cells, further diminishing isoagglutinin production [51]. However, further study is needed to prove the efficacy and safety of bortezomib combined with rituximab in the desensitization protocol for ABOi ALDLT.

Moreover, our review of studies suggested that there was no significant difference between the ABOi and ABOc groups for 1-year and 3-year OS and DFS for patients with HCC following ABOi ALDLT. However, the MELD score and the maximum tumor diameter were significantly lower in ABOi ALDLT for patients with HCC, probably because of the careful patient selection for ABOi ALDLT. Both the studies reporting on the recurrence of HCC for ABOi ALDLT revealed that rituximab does not increase the risk of HCC recurrence [25, 26]. Nevertheless, it has been found that overexposure to tacrolimus and basiliximab during the first year after LT increases the risk of HCC recurrence [52, 53].

**Figure 5:**
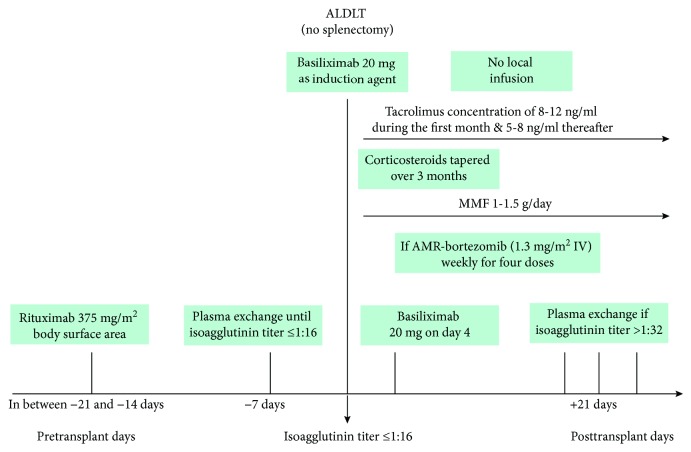
Suggested simplified desensitization protocol for ABO incompatible adult living donor liver transplantation. ALDLT—adult living donor liver transplantation; MMF—mycophenolate mofetil; AMR—antibody-mediated rejection.

Despite the high quality of the papers incorporated into this meta-analysis, there are various shortcomings concerning this meta-analysis. Firstly, there is a potential publication bias, because studies are less likely to outline negative findings. It could also be affected by the limited resources to identify unpublished trials. Secondly, only English-language studies were incorporated. Thus, the quality of outcomes was compromised to some extent, which is a typical reason for publication bias. Additionally, we could not identify two-arm studies comparing ABOi and ABOc ALDLT before the era of rituximab except for a few case reports or a one-arm study; this would have been of great importance if the comparative results before and after the era of rituximab were established. Moreover, the studies included in this meta-analysis have used different desensitization protocols and immunotherapies after LT; thus, it was difficult to harmonize these different protocols to the results of the meta-analysis. However, this meta-analysis is still of great significance for comparing different outcomes between ABOi and ABOc ALDLT in the era of rituximab and may prove beneficial for the clinicians in choosing the appropriate strategy (Figure 5).

Our meta-analysis included the largest number of studies comparing ABOi and ABOc ALDLT and all those using rituximab prophylaxis for ABOi ALDLT. ABOi ALDLT showed comparable results with that of ABOc ALDLT. However, CMV infection, biliary stricture, and AMR remain the major concerns in the era of rituximab. Nevertheless, a clinical trial is required for the comparisons of patient outcomes with/without rituximab, dosing, and timing of rituximab in a large cohort; anyhow, it would be hard to withdraw rituximab prophylaxis when the current outcomes are so much promising in the era of rituximab. Thus, we suggest the need for an effective and standardized desensitization protocol in addition to rituximab in the future.

## Figures and Tables

**Table 1:** Study characteristics included in meta-analysis.

Study ID	Study	LT type	Country and institute	Study period	Study type	Arms	No. of pts.	Disease characteristics	Follow-up	DZ protocol	Immunosuppressant	Nos.
1	Kim JM et al. (2018) [25]	Adult LDLT	South Korea, Samsung Medical Center	2010 to 2015	Retrospective	ABOi	59	HCC	28 ± 19 months	Rituximab+TPE	Induction: basiliximab+PGE1+gabexate mesilate+methylprednisolone. Maintenance: corticosteroids+tacrolimus+MMF	9
						ABOc	181		31±19 months			
2	Yoon et al. (2018) [26]	Adult LDLT	South Korea, Asan Medical Center	2008 to 2015	Retrospective	ABOi	165	HCC	48 months	Rituximab+TPE+LGIT	Maintenance: corticosteroids+tacrolimus+MMF	9
						ABOc	753		48.7 months			
3	Song WG et al. (2016) [27]	Adult LDLT	South Korea, Asan Medical Center	2008 to 2013	Retrospective	ABOi	235	HBV, HCV, ALF, cirrhosis, and ACLF	34 ± 13.3 months	Rituximab+TPE+LGIT±splenectomy±cyclophosphamide	Maintenance: corticosteroids+tacrolimus+MMF+basiliximab	8
						ABOc	1301		34 ± 13.3 months			
4	Kim JM et. al (2016) [28]	Adult LDLT	South Korea, Samsung Medical Center	2010 to 2013	Retrospective	ABOi	47	HBV, HCV, ALF, HCC, alcoholic, cirrhosis, and ACLF	25 ± 11.5 months	Rituximab+TPE±LGIT	Maintenance: corticosteroids+tacrolimus+MMF+basiliximab	7
						ABOc	94		23 ± 7.5 months			
5	Kim JD et al. (2016) [29]	Adult LDLT	South Korea, Catholic University of Daegu	2011 to 2014	Retrospective	ABOi	25	HBV, HCV, ALF, HCC, and ACLF	22.6 ± 17.2 months	Rituximab+TPE+LGIT+MMF	Maintenance: corticosteroids+tacrolimus+MMF	7
						ABOc	75		22.6 ± 17.2 months			
6	Kim SH et al. (2018) [22]	Adult LDLT	South Korea, National Cancer Center	2014 and 2016	Retrospective	ABOi	43	HBV, HCV, ALF, HCC, cirrhosis, and ACLF	20.9 ± 7.9 months	Rituximab+IVIG	Induction: basiliximab Maintenance: corticosteroids+tacrolimus+MMF	7
						ABOc	86		21 ± 5.6 months			
7	Lee CF et al. (2015) [30]	Adult LDLT	Taiwan, Chang-Gung Memorial Hospital	2006 to 2013	Retrospective	ABOi	46	HBV, HCV, ALF, HCC, alcoholic, cirrhosis, PBC, and ACLF	>5 years	Rituximab±TPE	Maintenance: corticosteroids+tacrolimus+MMF	8
						ABOc	340		>5 years			
8	Ikegami T et al. (2016) [31]	Adult LDLT	Japan, Kyushu University Hospital	1997 to 2013	Retrospective	ABOi	19	ALF, cholestatic disease, and cirrhosis	5.1 ± 2.1 years	Rituximab+TPE+LGIT+splenectomy±IVIG	Maintenance: corticosteroids+tacrolimus+MMF	8
						ABOc	389		5.1 ± 2.1 years			
9	Chae MS et al. (2018) [24]	Adult LDLT	South Korea, St. Mary's Hospital	2009 to 2016	Retrospective	ABOi	32	HBV, HCV, ALF, alcoholic, autoimmune, and cryptogenic	3.3 ± 1.02 years	Rituximab+TPE+LGIT	Induction: basiliximab Maintenance: corticosteroids+tacrolimus+MMF	9
						ABOc	32		3.3 ± 1.02 years			

ABOc—ABO compatible; ABOi—ABO incompatible; LGIT—local graft infusion therapy; MMF—mycophenolate mofetil; PGE1—prostaglandin E1; IVIG—intravenous immunoglobulin; TPE—total plasma exchange; NOS—Newcastle-Ottawa quality assessment scale; HBV—hepatitis B virus; HCV—hepatitis C virus; ACLF—acute-on-chronic liver failure; ALC—acute liver failure; PBC—primary biliary cirrhosis; DZ protocol—desensitization protocol.

## Abbreviations

ABOc: ABO compatible
ABOi: ABO incompatible
ACR: Acute cellular rejection
ALDLT: Adult living donor liver transplantation
AMR: Antibody-mediated rejection
CMV: Cytomegalovirus
ACR: Acute cellular rejection
HBV: Hepatitis B virus
HCC: Hepatocellular carcinoma
HCV: Hepatitis C virus
IHs: Isohemagglutinins
LDLT: Living donor liver transplantation
LT: Liver transplantation
MELD: Model for end-stage liver disease
AFP: alpha-Fetoprotein
TPE: Total plasma exchange
IVIG: Intravenous immunoglobulin G
DFS: Disease-free survival
OS: Overall survival.

## Supplementary Materials

Supplementary Materials Supplementary Table 1 Newcastle-Ottawa quality assessment scale. Supplementary Figure 1: forest plot of HCC patients. (a) AFP level, (b) MELD score for HCC patients, (c) maximum tumor diameter, (d) number of tumors, (e) 1-year overall survival for HCC patients, (f) 3-year overall survival for HCC patients, (g) 1-year disease-free survival, (h) 3-year disease-free survival, (i) 1-year disease-free survival beyond Milan criteria, and (j) 3-year disease-free survival beyond Milan criteria.
