# Supplementary material for: ABO-Incompatible Adult Living Donor Liver Transplantation in the Era of Rituximab: A Systematic Review and Meta-Analysis
Source: Gastroenterol Res Pract. 2019 Jun 11;2019:8589402. doi: 10.1155/2019/8589402 (PMC6594289; doi:10.1155/2019/8589402)
Supplement: Supplementary Materials — Supplementary Table 1 Newcastle-Ottawa quality assessment scale. Supplementary Figure 1: forest plot of HCC patients. (a) AFP level, (b) MELD score for HCC patients, (c) maximum tumor diameter, (d) number of tumors, (e) 1-year overall survival for HCC patients, (f) 3-year overall survival for HCC patients, (g) 1-year disease-free survival, (h) 3-year disease-free survival, (i) 1-year disease-free survival beyond Milan criteria, and (j) 3-year disease-free survival beyond Milan criteria. [file 8589402.f1.doc]

**Supplementary Table 1.** Newcastle-Ottawa quality assessment scale

|  | Selection | | | | Comparability | Outcome | | |  |
| --- | --- | --- | --- | --- | --- | --- | --- | --- | --- |
| Study | Representativeness of Exposed Cohort | Selection of Non exposed | Ascertainment of Exposure | Outcome Not Present at Start  Of study | Comparability of cohorts | Assessment of Outcome | Adequate Follow-Up Length | Adequacy of Follow-Up | Overall |
| Kim JM et. al (2018)^23^ | 1 | 1 | 1 | 1 | 2 | 1 | 1 | 1 | 9 |
| Yoon et. al (2018)^26^ | 1 | 1 | 1 | 1 | 2 | 1 | 1 | 1 | 9 |
| Song WG et. al (2016)^27^ | 1 | 1 | 1 | 1 | 2 | 0 | 1 | 1 | 8 |
| Kim JM et. al (2016)^28^ | 1 | 1 | 1 | 1 | 1 | 0 | 1 | 1 | 7 |
| Kim JD et. al(2016)^29^ | 1 | 1 | 1 | 1 | 1 | 0 | 1 | 1 | 7 |
| Kim SH et. al(2018)^22^ | 1 | 1 | 1 | 1 | 2 | 0 | 1 | 0 | 7 |
| Lee FC et. al(2015)^30^ | 1 | 1 | 1 | 1 | 2 | 0 | 1 | 1 | 8 |
| Ikegami T et. al(2016)^31^ | 1 | 1 | 1 | 1 | 1 | 1 | 1 | 1 | 8 |
| Chae MS et.al (2018)^25^ | 1 | 1 | 1 | 1 | 2 | 1 | 1 | 1 | 9 |

**Supplementary Figure 1.** Forest plot HCC Patients: 1A. **AFP level, 1B. MELD score for HCC Patients, 1C. Maximum tumor diameter, 1D. Number of tumors, 1E. 1 year Overall Survival for HCC Patients, 1F. 3 year Overall Survival for HCC Patients, 1G. 1 year disease free survival, 1H. 3 year disease free survival, 1I. 1 year disease free survival beyond Milan criteria, and 1J. 3 year disease free survival beyond Milan criteria.**

1A. **AFP level**

**
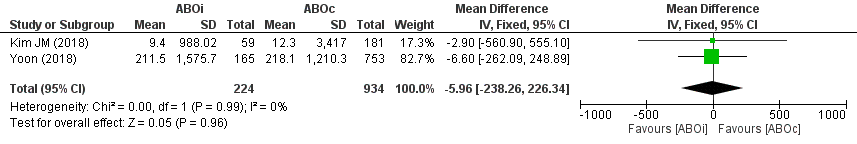
**

**1B. MELD score for HCC Patients**

**
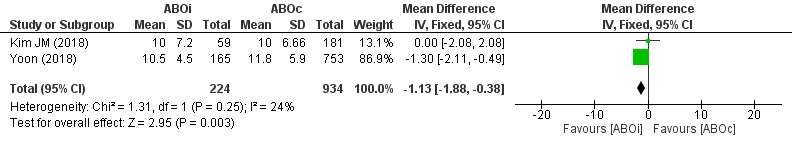
**

**1C. Maximum tumor diameter**

**
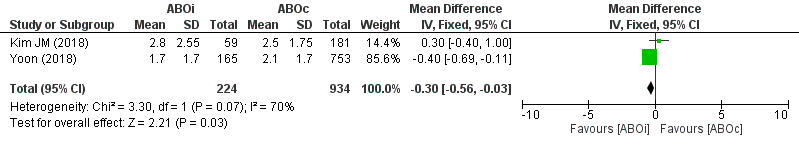
**

**1D. Number of tumors**

**
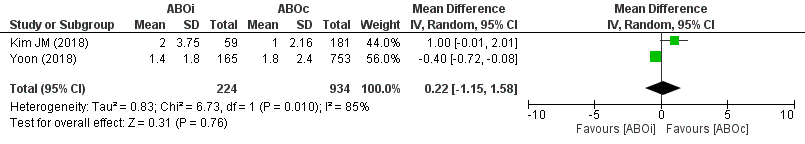
**

**1E. 1 year Overall Survival for HCC Patients**

**
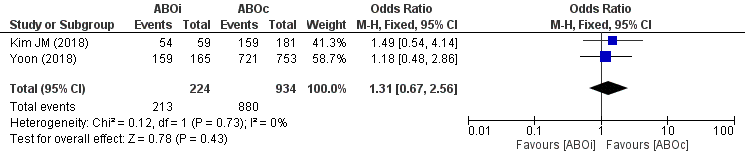
**

**1F. 3 year Overall Survival for HCC Patients**

**
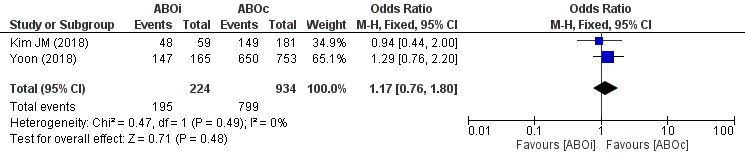
**

**1G. 1 year disease free survival**

**
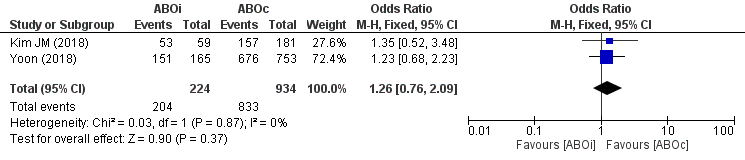
**

**1H. 3 year disease free survival**

**
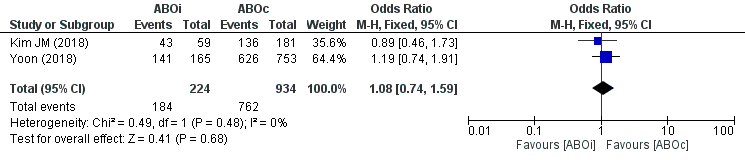
**

**1I. 1 year disease free survival beyond Milan criteria**

**
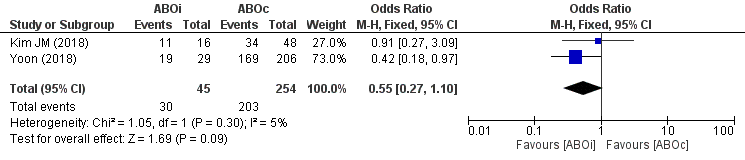
**

**1J. 3 year disease free survival beyond Milan criteria**

**
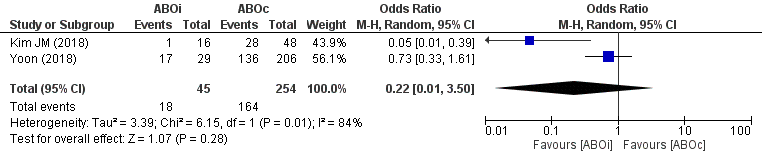
**
